# Recent Advances in pH-Responsive Liposomes: Lessons Learnt and New Directions in Nanomedicine Development

**DOI:** 10.3390/ma18184295

**Published:** 2025-09-13

**Authors:** Antonia Georgia Tsirogianni, Maria Chountoulesi, Natassa Pippa

**Affiliations:** Section of Pharmaceutical Technology, Department of Pharmacy, School of Health Sciences, National and Kapodistrian University of Athens, Panepistimioupolis Zografou, 15771 Athens, Greece; angetsirogianni@gmail.com

**Keywords:** pH-responsive, liposomes, anticancer, triggered release, phospholipids, gene therapy, acid microenvironment, tumor targeting

## Abstract

The development of pH-responsive liposomes represents a promising strategy for targeted drug delivery, enhancing therapeutic efficacy while minimizing toxicity and side effects. These innovative nanocarriers are typically synthesized via the thin film hydration method and can be further modified to exhibit stimuli-responsive behavior. pH-responsive liposomes are biocompatible, biodegradable, and capable of encapsulating both hydrophilic and hydrophobic drugs, increasing their versatility in drug delivery. In this mini-review, we explore the mechanism of action of pH-responsive liposomes, analyzing the factors that influence both their intracellular and extracellular behavior. Various formulations are examined, and their characteristics are compared to optimize therapeutic outcomes. Furthermore, we discuss the potential applications in anticancer therapy, in gene therapy, and in bacterial infections as vaccines and diagnostic agents.

## 1. Introduction

Liposomes are advanced nanotechnology-based drug delivery systems that have revolutionized the way therapeutic agents are transported and delivered within the body. These amphipathic spherical vesicles, composed of phospholipid bilayers, have the ability to encapsulate both hydrophilic and hydrophobic drugs, allowing them to serve as versatile carriers for a wide range of therapeutic agents [1]. Liposomes have gained significant attention in fields such as oncology, immunology, and infectious disease treatment due to their biocompatibility and their ability to protect drugs from degradation, all of which improve drug solubility and bioavailability. Despite these considerable advantages, conventional liposomes still face several limitations that hinder their full potential in drug delivery. A key challenge is the lack of precise targeting, where drugs may not accumulate at the specific site where they are needed. Without targeting, therapeutic agents can be distributed across non-targeted tissues, which can drastically reduce the effectiveness of treatment. Additionally, conventional liposomes can be rapidly cleared from the bloodstream by the immune system, reducing the circulatory time and limiting their therapeutic effect. In response to these challenges, scientists have developed innovative stimuli-responsive liposomal formulations with mechanisms that enable controlled drug release upon exposure to specific environmental triggers [2]. These liposomes can be activated by specific environmental triggers such as pH changes, temperature variations, redox conditions, enzyme activity, or external stimuli like UV light and ultrasound, exhibiting improved targeting and controlled release mechanisms [3].

**Scheme 1:**
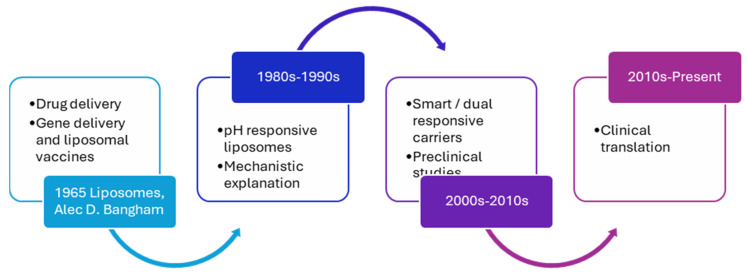
Historical overview of pH responsive liposomes.

Historically, in the late 1970s, after a fifteen-year-long observation of the “lipidic bubbles” by Alec D. Bangham, pH-responsive and other stimulus-responsive liposomes appeared in the literature (Scheme 1) [1,2,3]. The historical overview is presented in Scheme 1. They have attracted considerable interest in their ability to enhance therapeutic efficacy while reducing off-target effects thanks to their advanced mechanism of action. These specialized liposomes take advantage of the acidic microenvironment usually found in tumors or inflamed tissues, allowing for site-specific drug release. The tumor microenvironment, in particular, is inherently acidic due to the Warburg effect, where cancer cells exhibit increased metabolic activity, glycolysis, and lactic acid production. As a result, the extracellular pH in tumors falls to around 6.5–6.8, in contrast to the normal pH of healthy tissues, which is around 7.4. The pH difference plays a crucial role in the design of pH-triggered liposomes, which are engineered to remain stable in normal physiological conditions, preventing premature drug release during circulation, but when they encounter the acidic environment of a diseased tissue, their structure changes. Their operational mechanism relies on the protonation and deprotonation of functional groups in their structure. When exposed to the acidic conditions in tumor tissue, the protonation of carboxyl groups within the liposomal membrane occurs, which alters the liposome’s chemical properties to be more lipophilic, causing it to collapse [4,5]. The mechanistic explanation of the functionality of pH-responsive liposomes was very important for the development and synthesis of both fusogenic lipids and pH-sensitive polymers of various architectures. After the 2000s, the revolution of the “smart” nanocarriers is synonymous with stimulus-responsive carriers, where the pH is the main trigger for the alteration of the physicochemical and morphological characteristics of the liposomes (and/or other nanocarriers), leading to drug release. In broad terms, low pH levels trigger the membrane’s destabilization and fusion, eventually leading to controlled drug discharge at the targeted site. Such a controlled release mechanism enhances drug accumulation in diseased tissues while significantly lowering the risk of systemic exposure to healthy cells. The biocompatibility, biodegradability, and reduced immunogenicity make them a superior alternative to traditional treatments [6]. As a result, their applications extend to diverse areas, including their use as nanovesicles for cancer drug delivery, vaccine carriers for bacterial infections, and agents to eliminate biofilm-based infections. Furthermore, they can also be utilized in food preservation to prevent the deterioration of fruits and vegetables. The structural design of pH-responsive liposomes can be further modified to enhance their precision and efficacy. Researchers are investigating the development of dual-responsive liposomes that respond to both pH and redox conditions or enable triggered drug release in response to pH changes combined with UV exposure or ultrasound stimulation. Several generations of liposomes have been designed, approved for clinical treatment, and actively progressed towards the market, making a groundbreaking advancement in medicine.

The aim of this mini-review is to present the advantages of pH-responsive liposomes and to highlight their added value in the field of pharmaceutical technology. Their usage for delivery of antitumor Active Pharmaceutical Ingredients (APIs) makes them ideal drug delivery platforms by minimizing the adverse reactions, leading to an increased therapeutic index. Special attention is also given to other applications in the field of nanomedicines, such as vaccine development and infectious diseases.

## 2. Methods

**Scheme 2:**
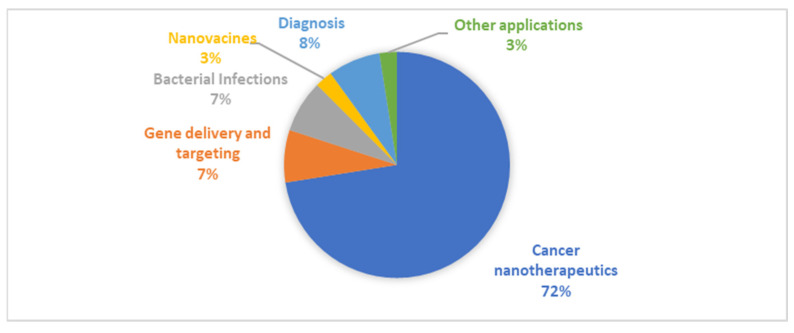
Statistical analysis of the applications of pH-responsive liposomes according to the research criteria of this study.

The literature search was conducted in the PubMed and Google Scholar databases using the keywords “pH-responsive liposomes” between 2020 and 2025 (the total number of papers is 51) (Scheme 2). References used for the review and statistical analysis of the applications of the pH-responsive liposomes were between 5 and 56.

## 3. Recent Advances in pH-Responsive Liposomes

These liposomal formulations can be classified based on their application and the type of drug or active compound they encapsulate, as different therapeutic agents require specific formulations for optimal efficacy. These categories include chemotherapeutic or other drugs for cancer treatment, gene and RNA therapeutics for targeted genetic interventions, antibiotics for bacterial infections, immunogenic agents for vaccine development, and diagnostic agents.

### 3.1. Structural Properties of pH-Responsive Liposomes

#### 3.1.1. Formulation Approaches

First, a more in-depth investigation into the mechanism of action of pH-sensitive liposomes is required and has been conducted by numerous researchers exploring various factors that could influence the behavior of these liposomes in targeted environments. Ashrafizadeha et al. used various examples of different APIs, such as doxorubicin, siRNA, SN25860, oleanolic acid, and ursolic acid, encapsulated in liposomes with a high entrapment efficacy, and demonstrated rapid drug release in the tumor’s acidic environment and efficient endosomal escape. Common materials used for the synthesis of each liposomal formulation include peptide H7K(R2)2, PEGylated lipids, OA, HA, folate, glycidol, and malachite green leuco derivatives (MG-Xs). These liposomes are proposed for treating multiple cancer types, including gliomas, colorectal tumors, and cervical, pancreatic, and breast cancer cells [5]. Antoniou et al. also investigated different formulations of pH-sensitive liposomes in anticancer therapy, aiming to enhance drug delivery while minimizing toxicity. Several drugs were used either individually or for the codelivery of both hydrophilic and hydrophobic drugs.

#### 3.1.2. Influence of Lipids and PEG

Liposomes were developed using the thin film hydration method with materials such as Dioleoylphosphatidylethanolamine (DOPE), Cholesteryl hemisuccinate (CHEMS), Distearoylphosphatidylethanolamine DSPE, dipalmitoylphosphatidylethanolamine DPPE, EYPC, Dipalmitoylphosphatidylcholine DPPC, cholesterol (CHOL), PC, PE, PEG-phospholipids, Hz bonds, and several polymers and copolymers. Additionally, various peptides (CPP, cRGDfK, lysine, cysteine, SKDEEWHKNNFPLSP), orthoester nucleoside lipids, and PEPC were used. Nevertheless, the interactions with serum proteins have not yet been studied [4]. A similar approach was pursued by Rana et al., who analyzed the mechanism of action of pH-responsive liposomes in anticancer therapy. By examining several formulations composed of different materials such as PC, CHOL, PE, PEGylated lipids, polymers, or Hz constructed and formed using the thin film hydration method, they underlined its potential for inhibiting tumor development, decreasing the growth of breast, cervical, and prostate cancer cells, or HeLa, M109, 4T1, and L929 cell lines [7]. In a closer scrutiny, Kashapov et al. analyzed how pH-sensitive lipids destabilize the liposomal membrane, triggering drug release. These pH-responsive lipids and surfactants undergo protonation in acidic conditions, causing liposome collapse [6]. Furthermore, Hassan Shah et al. used DOPE, CHEMS, and DSPE-PEG2000 in different molar ratios to synthesize pH-sensitive liposomes via the thin film hydration method and compared the resulting formulations. They observed that the encapsulation efficacy decreased as the concentration of DOPE increased, highlighting PL3 (55:4:05) as the more suitable composition. This formulation had a particle size of approximately 190 nm, a negative zeta potential, and a narrow Polydispersity Index PDI. The liposomes, loaded with cisplatin (CDDP), demonstrated pH-triggered drug release following the Korsmeyer-Peppas model, achieving more than 80% release at acidic pH while less than 40% was released at basic pH conditions. A limitation of this study is the lack of in vivo experiments to further evaluate the therapeutic efficacy of the pH- responsive liposomes [8].

#### 3.1.3. Encapsulation Efficiency and Trends

In a more profound study into liposomal formulation, Naziris et al. analyzed the physiochemical characteristics of pH-responsive chimeric liposomes. In order to evaluate the lyotropic and thermotropic behavior, size and ζ-potential, polydispersity, pH responsiveness, and protein interactions, they developed liposomes using HSPC, PDMA-b-PLMA (70–30 {1} and 58–42 {2}), and (9:0.1 and 9:0.5) via the thin film hydration method. These formulations displayed size ranges (Dh = 75.4 to 189.1 nm) and ζ-potential variations (−0.27 to 19.3 mV) corresponding to dispersion medium, biomaterial concentration, and pH adjustment. The liposomes showed high stability, low PDI, pH-triggered drug release, and low in vivo toxicity, indicating their potential for clinical applications as drug delivery systems. However, further studies are required for scale-up and clinical trials [9]. Rustad et al. investigated the role of PEG chain length in liposomes designed for cancer treatment. They synthesized pH-responsive PEGylated liposomes using DOPE, CHEMS, and DSPE-PEG750 or/and 2000 (6:4:0.1) in a chloroform-methanol (50:50) solution via the thin film hydration method. The liposomes encapsulated the model marker calcein, and both formulations exhibited increased calcein uptake, indicating that neither PEG nor its chain length affected the uptake. The liposomes had small sizes, negative surface charges, and narrow PDI. Release profile analysis revealed that liposomes comprising longer PEG chains were more stable and less responsive to pH alterations, which was later confirmed as pH-Lip-PEG2000_C exhibited significantly less calcein, release highlighting the stabilizing effect of the longer PEG chain compromising the pH responsiveness. Despite proving valuable insights, further research is needed to assess different PEG concentrations and validate findings through in vivo experiments [10]. In the pursuit of understanding how various factors alter liposomal formulation, Yanagihara et al. investigated how liposome size and cholesterol content affect the drug release and stability in pH-responsive liposomes modified with MGlu-Aquaβ. Liposomes were prepared via thin film hydration method with EYPC, CHOL (100:0, 80:20, 50:50, mol/mol), DPPC, and CHOL (50:50, mol/mol), followed by extrusion through membranes of varying pore sizes (1000, 400, 200 nm). Their findings highlighted that incorporating cholesterol into EYPC liposomes enhanced stability, with the optimal ratio being 80:20. Small liposomes exhibited higher drug release, especially in acidic environments. Unsaturated EYPC-based liposomes demonstrated greater suitability compared to DPPC-based ones due to increased membrane flexibility. Despite promising results, long-term stability remains a limitation, necessitating further research for clinical application [11].

Lado-Touriño et al. investigated the role of (F)2-(imidazol-1-yl)succinic acid (ISUCA), pH-sensitive lipids incorporated in liposomes. For this purpose, they used PE, PC, PS, or OA, Palmitoyl-oleoyl-phosphatidylcholine POPC, and ISUCA to form liposomes with ISUCA, ISUCA-2Ol, ISUCA-2Pal, and ISUCA-Pal-Ol through coarse-grained molecular dynamics simulations (MARTINI forcefield). Their mechanism of action focuses on destabilizing the liposomal bilayers under acidic conditions, enabling pH- responsive drug release. In greater detail, these lipids have the ability to increase the area per lipid and diffusion coefficients while decreasing hydrophobic thickness and the order parameter. Furthermore, chemically, ISUCA functions as a hydrophilic head group that becomes protonated in acidic environments. This protonation destabilizes the liposomal membrane, triggering drug release, following the mechanism of action of pH-responsive liposomes. Additional research exploring their stability in physiological environments, biocompatibility, and interactions with blood proteins should be conducted [12]. The pH-dependent way O-methyl-serine dodecylamine hydrochlorideMSDH affects liposome behavior was also studied by Villamil Giraldo et al. MSDH is a lysosomotropic detergent that forms liposomes at physiological conditions but destabilizes them in acidic pH, leading to the release of encapsulated drugs. In addition, several adjustments to the liposome surface were analyzed [13]. More thoroughly, Fuentes et al. used 5,10,15,20-tetrakis(4-trimethylammoniophenyl)porphyrin (TTMAPP), a cationic O_2_ sensitizer, in liposomes as a different approach to the treatment of human prostate cancer cells, which was adsorbed onto an anionic silica surface and encapsulated into liposomes. The novel formulation of the liposomes with TSPP and DOPC, prepared through the lipid film hydration method followed by centrifugation, exhibited pH-sensitive release behavior with higher release rates observed at acidic pH (80% at pH = 2.3). A significant drawback of this formulation is its concentration-dependent cytotoxicity. Future research should aim to refine the formulation to reduce adverse effects while maintaining efficacy [14]. Another example is the research made by Goldbach et al., who developed pH-responsive bundles-2 (pRO-2) I56V, capable of incorporating into liposome surfaces and inducing their lysis. These bundles contain hydrogen bond networks with histidine residues, enabling pH-dependent disruption of the liposome membrane. As a result, the drug is released in the tumor’s acidic environment, enhancing therapeutic efficacy while minimizing potential toxicity [15]. Alghurabi et al. introduced an innovative method for oral colonic drug delivery by incorporating a bile salt into a pH-responsive liposomal formulation. Researchers tested several molar ratios of DPPC, cholesterorol (CHOL), and sterilamine SA (7:3:0/7:3:0.25/7:3:0.5/7:3:1/7:3:2/7:3:3/7:3:4/8:2:3/6:4:3), and liposomes were prepared via the thin film hydration method followed by sonication or extrusion and further coated using the electrostatic layer-by-layer technique to encapsulate budesonide (0.25). The resulting formulations exhibited large particle sizes, a low polydispersity index, and negative ζ-potential with uniform and efficient coating. Their kinetics profile revealed pH-dependent drug release behavior, with higher drug release at increasing pH levels. Among the tested formulations, the 7:3:1 molar ratio demonstrated higher positive surface charge, minimum aggregation, and highest encapsulation efficiency. Researchers also concluded that sterilamine’s concentration did not negatively impact size or stability; however, further research is required for using 3 as the molar ratio of this material [16]. Lastly, regarding the inner liposomal core, the remote loading of a hydrophobic compound into the aqueous core of liposomes was evaluated by Odeh et al. by using a transmembrane pH gradient strategy. For this purpose, they used curcumin loaded into secondary and tertiary amino-modified β-cyclodextrins, which served as carriers. The liposomes were synthesized using the thin film hydration method by DPPC and CHOL (65:35). Using HSPC liposomes with an incorporated peptide, PDMAEMA-b-PLMA 1 or 2, in different molar ratios and dispersion mediums, they formed βCDs liposomes with diameters around 100 nm, PDI around 0.5, and ζ-potential around 15 mV. This novel formulation improved curcumin’s solubility, encapsulation efficiency, and bioavailability, highlighting its potential for targeted drug delivery and the necessity of in vivo investigation [17].

### 3.2. Recent Developments in pH-Responsive Liposomes for Applications in Pharmaceutical Nanotechnology

#### 3.2.1. Cancer Nanotherapeutics

pH-responsive liposomes are predominantly utilized in cancer treatment thanks to their unique mechanism of action, which reduces the risk of chemotherapeutic agents that are associated with high systemic toxicity. Several researchers have studied their formulations and potential use while making alterations to enhance their therapeutic efficacy. Nunes et al. developed folate-coated pH-sensitive liposomes for tumor-targeted drug delivery. The liposomes, composed of DOPE, CHEMS, and DSPE-PEG2000 (5.8:3.7:0.45:0.05) and DOPE, CHEMS, DSPE-PEG2000, and DSPE-PEG2000-Fol (5.8:3.7:0.45:0.05), were prepared using lipid film hydration and extrusion methods for size reduction. The prepared formulations showed sizes around 160 nm with low polydispersity and neutral z-potential. Encapsulating the anticancer drug irinotecan at high rates, around 60% (encapsulation efficiency EE = 62.3 ± 0.9%, loading capacity LC = 6.2 ± 0.1%) this formulation is proposed for colorectal cancer therapy. The authors proved controlled release in the tumor’s acidic environment, indicating higher release rates at lower pH. Further investigation is needed, as it shows potential for clinical trials in other types of cancer [18]. Another example is presented by Park et al., who prepared and evaluated FRβ-targeted pH-sensitive liposomes against non-small cell lung cancer (NSCLC). The liposomes were synthesized using the thin-film hydration technique and loaded with doxycycline and docetaxel (EE = 60.1 ± 1.1%, LC = 19.3 ± 3.6%) and formed a spherical structure with a diameter of approximately 80 nm and a neutral ζ-potential. Their composition (DPPC, DSPE-PEG-NH2, CHEMS, PEG bis(amine), and Folate (8.8:1.9:1)) enhanced the pH-dependent targeted release of drugs: 42% for docetaxel and 25% for doxycycline at pH = 7.4, 61% for docetaxel and 39% for doxycycline at pH = 6.5, and 99% for docetaxel and 78% for doxycycline at pH = 4.0. This is a promising formulation for early diagnosis and treatment of Non-small cell lung cancer (NSCLC), yet further studies are needed to explore its efficacy in metastasis [19]. In a similar vein, Pandey et al. designed pH-responsive liposomes with folic acid and iRGD surface modifications for breast cancer treatment. These liposomes were synthesized using the thin-film hydration method, incorporating phospholipids with 80% phosphatidylocholine (E80), DOPE, CHEMS (3.65:2.65, *w*/*w*), and DSPE-PEG2000-COOH. The resulting spherically shaped formulations had a diameter of approximately 150 nm, a ζ-potential of 15 mV, and a PDI of 0.22. They were loaded with 5-fluorouracil, achieving high rates of encapsulation efficacy and loading capacity. Drug release was evaluated at 24, 48, and 72 h at pH = 7.4 and 5.5, demonstrating pH-triggered drug release in acidic environments. Further optimization and preclinical development are required [20]. Furthermore, in another study, Alrbyawi evaluated internal-stimuli-responsive liposomes by analyzing various liposomal formulations targeting metastatic colorectal cancer and cervical cancer (HeLa) cells. While 5-Fluorouracil was a common drug component, he also examined liposomes containing imatinib, docetaxel, paclitaxel, or doxorubicin. The formulations consisted of lipids and phospholipids (CHEMS, CHOL, DSPE-PEG2000, DSPC, DOPE, Dioleoyltrimethylammonium propane DOTAP, HSPC, DPPG, PE, and OA), polymers and copolymers (SMA, sulfadimethoxine-based copolymer, 3-methylglutarylated hyperbranched poly(glycidol) polymer), targeting ligands (HA, sodium hyaluronate layers, folate-PEG3350-CHEMS), nucleic acids (siRNA), and aspartic acid. All formulations exhibited enhanced drug release at acidic pH; however, their effectiveness in targeted delivery to other specific sites was not explored [21]. García et al. designed a novel nanocarrier with a self-targeted component for both chemotherapy and photothermal therapy against breast and ovarian cancer cells. NH2-PEGylated gold nanoparticles were encapsulated at high efficiency (EE = 78 ± 4%) in liposomes composed of DPPC, CHOL, DDAB (75.24:3.35:21.42 and 45:40:15), and DG-CDP were synthesized through thin-film hydration and extrusion methods. The formulation exhibited promising characteristics, including a diameter around 650 nm, a slightly negative ζ-potential, and low dispersity. The drug demonstrated controlled release under acidic and hypothermia conditions following zero order, Peppas (with n values near to 0.5), or the Higuchi model. Its dual pH- and thermo-responsive behavior combined with the ability to evade immune system responses emphasize its capabilities. However, future studies should focus on its safety, efficacy, and comparison with alternative treatments in different experimental animal models [22]. Zhai et al. enhanced pH-responsive properties in a novel formulation by incorporating an acid-sensitive peptide (DVar7) into liposomes. The liposomes loaded with the anticancer drug doxorubicin at high efficiency, and injecting glucose to regulate acidity in the tumor microenvironment, they achieved enhanced therapeutic efficacy while minimizing toxicity. The formulation was synthesized via the thin film hydration method to obtain DSPE-PEG2000-DVar, incubated with DOX-loaded liposomes and purified by filtration. The final composition comprised DOPE, CHEMS, DSPE-PEG2000, and DSPE-PEG2000-DVar7, [Cy5.5] (54:40:4:2:1, *w*/*w*%) and demonstrated a diameter of 130 nm with a narrow PDI, indicating great stability. In vivo studies proved pH-triggered drug release in the tumor’s acidic environment (pH = 5.3), five times higher than at pH = 7.4. This approach has margin for progress, particularly for highly malignant tumors, and could benefit from other thermal methods, such as heat-induced acidification, to further enhance therapeutic efficacy [23]. Lastly, dos Reis et al. investigated the mechanism of intracellular drug release from pH- sensitive liposomes using cervical cancer HeLa cells. For this purpose, they developed pH-responsive liposomes using DOPE, CHEMS, and DSPE-PEG2000/HSPC (5.8:3.7:0.5) via the lipid film hydration method, extrusion, and ultracentrifugation. These liposomes encapsulated doxorubicin, forming SpHL-DOX and nSpHL-DOX. Compared to free-DOX, SpHL-DOX showed faster intracellular release in contrast to nSpHL-DOX but promoted sustained release when it was contrasted to free-doxorubicinDOX. Using chloroquine and E64d, the study revealed that the release mechanism was influenced by lysosome acidification and protease activity. Alongside, the role of autophagy is yet to be clarified [24]. In a unique approach, Grace et al. designed and synthesized cationic pH-responsive liposomes as a potential carrier for all-trans retinoic acid (ATRA) to enhance targeted therapy for lung cancer. Utilizing the thin-film hydration method followed by ultracentrifugation and extrusion, liposomes composed of Dioleoyltrimethylammonium propane DOTAP and cholesterol (5:4) were successfully generated, exhibiting a diameter of 231 nm and a slightly positive ζ-potential. A high encapsulation efficiency was achieved for ATRA at a dosage of 0.8 μmol, with the drug being released in a gradual and sustained manner. Simultaneously, the formulation demonstrated pH-responsive drug release where the amount of ATRA at pH = 6 was significantly higher than at pH = 7.4, confirming its potential for tumor-selective drug delivery. However, challenges remain in large-scale production, particularly in maintaining stability and efficacy, which have not yet been analyzed [25]. Furthermore, their structural properties and pH responsiveness suggest their potential as a highly effective carrier for gene therapy. In the context of glioblastoma, Naziris et al. developed pH-responsive liposomes as carriers for the antiglioma agent (TRAM-34) in the treatment of glioblastoma. Using Eggphospahtidylcholine EPC, Poly(N,N-dimethylaminoethyl methacrylate)-block-poly(lauryl methacrylate) PDMAEMA-b-PLMA 1, and PDMAEMA-b-PLMA 2 in different molar ratios, they compared the resulting formulations while investigating their mechanism of action in anticancer therapy, leading to an optimal ratio of 9:0.5. The techniques applied included reversible addition-fragmentation chain-transfer (RAFT) for PDMAEMA-b-PLMA and the thin-film hydration method for chimeric liposomes. The nanocarriers exhibited sizes around 150 nm, with one variant demonstrating a slightly larger hydrodynamic diameter and higher surface charge than the other; however, both showed homogeneous size distribution. TRAM-34 was encapsulated at high rates (62–73%) and was released in a controlled, pH-responsive manner, achieving near complete release in an acidic environment within an hour. In vitro and in vivo studies confirmed their ability to inhibit glioma cells, encouraging further exploration for therapeutic applications [26]. Some researchers have focused on modifying the liposomal membrane to improve its targeting capabilities and enhance treatment effectiveness. First of all, Han et al. formulated pH-responsive PEGylated liposomes as an alternative method for treating idiopathic pulmonary fibrosis. They synthesized liposomes with DOPE, DSPE-PEG2000, and CHEMS through the ammonium sulfate gradient method and then loaded pirfenidone using the calcium acetate gradient method, achieving high rates of encapsulation efficacy. The outcome formulation had a quasi-spherical morphology and displayed a diameter of approximately 107 nm, a ζ-potential around −74 mV, and a very low PDI. Additionally, the drug release profile exhibited a sustained release, reaching 80% in 24 h, with an increased release rate under acidic pH conditions. Lastly, they observed that adding PSLs could prolong the drug’s action time. However, further optimization should occur regarding PSL incorporation or dosage adjustments [27]. Alongside this, Cheng et al. combined liposomes with amphiphilic dendrimers to target the tumor microenvironment for antigen delivery and immune modulation. For this purpose, PC and 3-methylglutarylated-dextran residues (Mglu-Dex) were purchased, and the formulations were loaded with sorafenib and hemin. The findings were promising, although the pharmacokinetic profiles of the individual and combined formulations have not been fully explored yet. Therefore, preclinical and clinical studies are necessary to investigate their toxicity, and off target effects and to better understand their mechanism of action. Several concerns remain regarding production stability, storage protocol, appropriate transport methods, and scalability to the industrial zone [28]. Another breakthrough in liposome formulation was the incorporation of an inhibitor into its membrane. On this prospect, Quian et al. suggested the synthesis of pH-responsive liposome-coated carbonic anhydrase inhibitor (CAI)-loaded calcium carbonate nanoparticles (CaCO_3_/CAI@Liposome (CCL)) as a therapeutic approach in anticancer immunotherapy. Its innovative mechanism of action distinguishes it by causing calcium death and enhancing immune responses. Due to its formulation, DOPA and DSPE-PEG5000, and preparation methods, one-pot method and thin-film dispersion-membrane extrusion technique, the resulting liposome exhibited pH-triggered CAI and Ca^2+^ release with nearly double the rate at acidic pH compared to alkaline conditions. The formulation loaded with the carbonic anhydrase inhibitor (CAI) achieved encapsulation efficacy around 60% and loading capacity approximately 8%. The results indicate the need for optimization to achieve higher EE rates by altering the small size (Dh = 116 nm) or the molar ratio. Further investigation should also be conducted into its potential use in other types of cancer [29]. Yao et al. encapsulated the API RA-V (2.68) PD-A/PD-L1 inhibitor (BMS-202) (2.15) in liposomes, making them capable of camouflage as cancer cells, which could be used in colorectal cancer therapy. In this regard, they used CHEMS, DSPE-mPEG2000-COOH, and DOPE (12.6:3.2:34.74, *w*/*w*), and through thin film hydration, ultrasonic treatment, and extrusion methods, they synthesized this novel formulation of large size (Dh = 192.46 ± 7.91 nm) and slightly negative ζ-potential. They noted high encapsulation efficacy (EE = 80.1% (for RA-V), EE = 79.6% (for BMS)) and controlled drug release in intracellular acidic environments. This method holds great potential for application in other types of cancer [30]. In another study, the researchers focused on modifying pH-responsive liposomes in a way to influence their mechanism of action. For instance, Shen et al. investigated pH- and photo-dual-responsive liposomes for targeted anti-tumor activity. Using the thin film ultrasonic dispersion method, liposomes were synthesized consisting of lecithin and the polymer PEG-OE-L in various molar ratios (100:0, 95:5, 90:10, 85:15, 80:20, *w*/*w*%), identifying 15% PEG-OE-L as the optimal concentration. Chlorine e6 (Ce6), paclitaxel, and doxorubicin were successfully entrapped, achieving high encapsulation efficiency (90%) and a loading capacity below 10%. The resulting liposomes formed small, spherical particles with a negative ζ-potential. The drug release profile revealed rapid release and enhanced endocytosis in a slightly acidic environment and under 660 nm light irradiation. Without light exposure, release was moderate but increased significantly at lower pH, while laser irradiation further accelerated it. However, further in vivo studies are essential to confirm the efficacy and the safety of this formulation [31]. In this context, Qi et al. explored (7-ethyl-10-hydroxycamptothecin)-SN38-based drug delivery systems for anticancer therapy with prolonged, pH-dependent drug release behavior due to reversible pH-dependent hydrolysis of the lactone ring. At pH = 7.4, the lactone ring partially hydrolyzes into an inactive carboxylate form, while at pH = 9 it fully converts to the carboxylate form. The formulations investigated included SN38-PA, LA-SN38, and Di-SN38-PC liposomes; irinotecan-loaded liposomal nanoparticles (MLP); and transferrin (Tf) liposomes co-loaded with TAT-PEG-SN38 and surviving siRNA. They were tested on colon cancer, MCF-7, HBL-100, HT-29, and HeLa cells correspondingly, showing improved cytotoxicity, biocompatibility, and solubility, achieving longer blood circulation. However, the lack of clinical trials highlights the need for in vivo models to identify the differences between mouse tumor models and clinical patient tumors [32]. In a different approach, Tang et al. investigated a novel formulation of pH-responsive liposomes to demonstrate a synergistic strategy for anticancer therapy. The liposomes, composed of cholesterol, soy lecithin, DSPE-PEG2000, C18-TAT peptide, and CA (30:60:2:6:20), were prepared using the self-emulsifying solvent evaporation and thin film hydration methods. They exhibited a diameter of 155 nm, a ζ-potential of −22 mV, and a small PDI. Incorporating the Pt (IV) pro-drug disuccinatocisplatin (DSCP) and cinnamaldehyde (CA), this formulation enhanced intracellular reactive oxygen species production, triggering apoptotic pathways in tumor cells. Notably, drug release studies confirmed CA’s and DSCP’s pH-triggered release behavior in the tumor’s acidic microenvironment. However, these findings lack in vivo studies, particularly regarding cytotoxicity and bioavailability [33].

**Figure 1:**
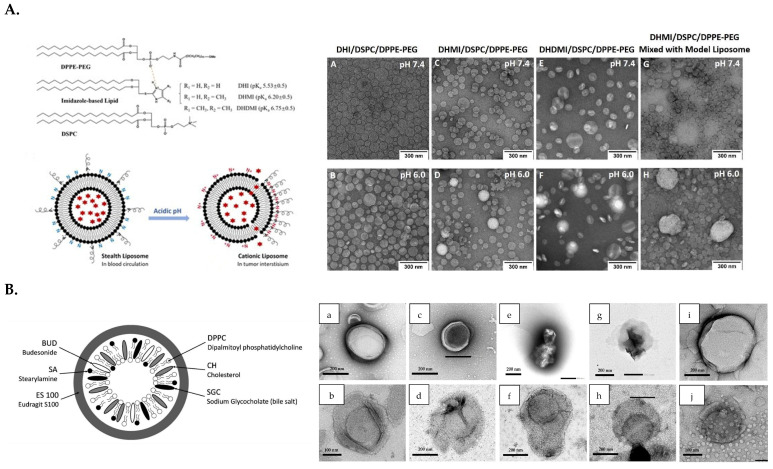
Characteristic surface and morphology alterations of the pH-sensitive liposomes during the pH-response process, triggering content release. (**A**) Imidazole-based convertible liposome and the respective TEM images in pH = 7.4 and pH = 6. Adapted from [34]. (**B**) Eudragit S100-Coated Bile Salt-Containing Liposomes and the respective TEM images in different pH media simulating the different gastrointestinal pH conditions(TEM images (a) SGC-Lip in SGF, (b) ES100-SGC-Lip in SGF, (c) SGC-Lip in FaSSIF, (d) ES100-SGC-Lip in FaSSIF, (e) SGC-Lip in FeSSIF, (f) ES100-SGC-Lip in FeSSIF, (g) Lip in FeSSIF, (h) ES100 -Lip in FeSSIF, (i) SGC-Lip in PBS pH 7.4, (j) ES100-SGC-Lip in PBS pH 7.4 after 2-h incubation at 37 °C). Adapted from [16].

Huang et al., trying to enhance the activity of liposomal drugs against solid tumors, prepared three novel lipids that carry imidazole-based headgroups of incremental basicity that were incorporated into the membrane of PEGylated liposomes containing doxorubicin (DOX) to render pH-sensitive convertible liposomes. The imidazole lipids were designed to protonate and cluster with negatively charged phosphatidylethanolamine-polyethylene glycol when pH decreases from 7.4 to 6.0, thereby triggering enhanced DOX release (Figure 1). The liposomes with the imidazole lipid of medium basicity showed the highest anticancer activity in 3D MCS [34].

**Figure 2:**
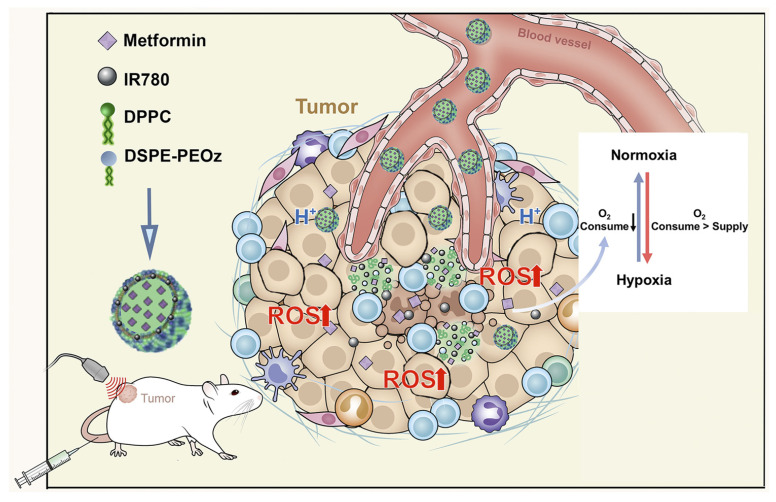
Schematic illustration of self-synthesized MI-PEOz-lip and its proposed anti-tumor mechanism. After intravenous injection, MI-PEOz-lip is expected to accumulate in tumors via the EPR effect. Then, the incorporation of pH-sensitive lipids into this liposome allows metformin to be rapidly released at the tumor site in response to the acidic pH of the tumor microenvironment. The released metformin reverses hypoxia in the tumor by reducing oxygen consumption and further increases IR780 to produce more ROS in hypoxic tumors. Adapted from [34].

pH-responsive liposomes were also designed, aiming to address various challenges in cancer therapy, including enhancing the efficiency of sonodynamic therapy (SDT) and reducing systemic toxicity. A major drawback of SDT is its reduced efficacy in the hypoxic tumor microenvironment as an oxygen-dependent therapy. Zhang et al. proposed a formulation composed of DPPC, cholesterol, and DSPE-PEOz2000 (12:4:4) that reduces oxygen consumption and attenuates hypoxia-induced resistance to SDT. The liposomes were prepared using the thin film hydration method and were loaded with an API (IR780) as a sonosensitizer and metformin, an antihyperglycemic drug, as the SDT enhancer. They exhibited a diameter of approximately 200 m, a negative surface charge, and were well-dispersed and homogeneous (Figure 2). The release behavior of both drugs was studied, demonstrating metformin’s pH-triggered release of up to 95% at pH = 5.5, while IR780 displayed slightly accelerated release at more acidic pH. This strategy for breast cancer treatment is considered a promising approach for potential clinical trials, where it should be evaluated whether individuals with liver failure should avoid it [35].

**Figure 3:**
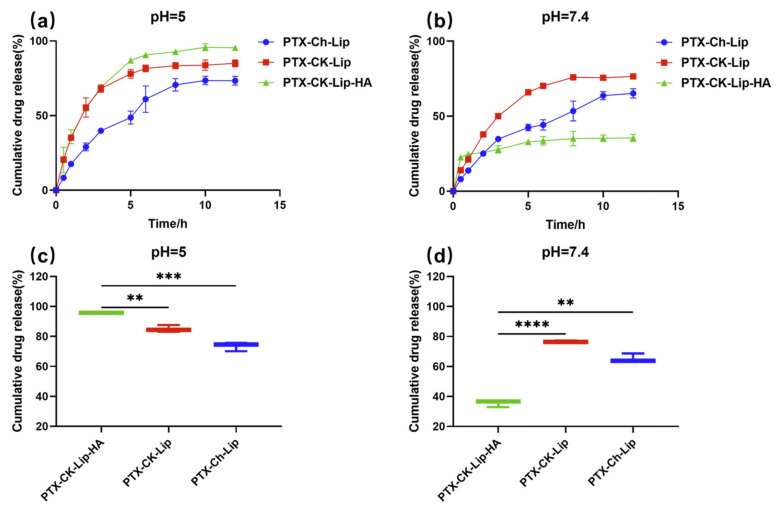
The cumulative drug release of liposomes in (**a**) pH = 5.0 and (**b**) pH = 7.4 PBS at 37 °C from 0 to 12 h. The cumulative drug release of liposomes in (**c**) pH = 5.0 and (**d**) pH = 7.4 PBS at 37 °C at 12 h. (n = 3; mean ± SD), ** *p* < 0.01, *** *p* < 0.001, and **** *p* < 0.0001. Adapted from [37].

Similarly, a concerning complication with dermaseptin-PP (65) in anticancer lung therapy is the elevated risk of hemolysis. Wang et al. addressed this by developing a novel formulation to encapsulate this a-helix anticancer peptide, aiming to enhance efficacy and reduce the risk of hemolysis. Using the thin-film hydration method, liposomes composed of cholesterol, DSPE-Hyd-PEG2k (25:10), lecithin, and DSPE were constructed with a diameter of approximately 97 nm, a ζ-potential of 7 mV, and a PDI of 0.15, indicating uniformity in size distribution and stability. The drug was loaded, achieving adequate encapsulation efficiency and loading capacity (EE = 86%, LC = 7%). Notably, cellular uptake increased significantly at pH 5 compared to pH 7.4, highlighting the system’s potential for targeted drug release in acidic tumor environments. The only limitation that requires further investigation is scalability [36]. The idea of incorporating a natural compound into the liposomal formulation persuaded You et al. to design liposomes with pH-responsive behavior modified with ginsenoside compound K(CK) and hyaluronic acid (HA). Liposomes composed of lecithin (egg yolk) and CHOL (100:30) were prepared via the thin film dispersion technique and encapsulated paclitaxel for targeting HCT-116 cells, obtaining exceptional encapsulation efficiency. The resulting formulations exhibited large particle sizes, low polydispersity index, and negative zeta potential, ensuring stability and targeted delivery. Drug release followed a pH-triggered mechanism, reaching nearly 100% release in acidic pH (Figure 3). Generally, kinetic modeling suggested Higuchi or Korsmeyer–Peppas behavior, with rapid release within the first 5 h, followed by a slower release from the 5th to the 10th hour, and ultimately stabilizing at the 12th hour. Further investigation into cellular uptake mechanisms is required along with in vivo animal studies to validate these promising findings [37].

Through another groundbreaking approach, Liu et al. introduce pH-sensitive azidosugar liposomes coated with a natural cancer-cell membrane, offering a novel approach to tumor-selective glycan engineering. These nanoparticles were formulated with DSPE-PEG-NH2, DOPE (1:1), and N-azidoacetylgalactosamine tetraacylated (Ac4GalNA), via the thin film hydration method. With a small size (Dh = 164 nm) and negative ζ-potential, they effectively encapsulated rhodamine B and exhibited pH-dependent release. Thoroughly, the drug’s release rate nearly tripled at an acidic pH compared to a basic pH after 12 h. These characteristics enhance tumor selectivity by reducing protein corona formulation and promoting efficient phagocytosis by macrophages, highlighting their potential as extracellular vesicles. However, concerns remain regarding the possibility of off-target effects on cancer cells, especially if some subtypes share the same biomarkers. Limitations associated with biopsies or surgical resections, such as patient discomfort and increased healthcare costs, persist [38]. Lastly, another research team focused on loading natural compounds into pH-sensitive liposomes to target the tumor’s acidic microenvironment. Lin et al. addressed the design of a formulation capable of loading and releasing cytotoxin (CTX) from cobra venom. For this purpose, they synthesized pH-responsive liposomes composed of DOPE, CHEMS, DSPC, and mPEG2000-DSPE (32:8:34:5, *w*/*w*) through the thin-film hydration method. The resulting nanoparticles exhibited a diameter of approximately 130 nm with a negative surface charge. The encapsulation efficacy or loading capacity may not be sufficiently high; however, CTX demonstrated sustained and controlled release within the tumor environment, achieving nearly 100% at pH = 5.5 after 72 h. These findings demonstrate great potential; nevertheless, further optimization is necessary. The PDI values are not adequately low, indicating the formulation’s instability, which could lead to aggregation, especially during extended storage periods of up to 30 days [39]. Additional work in cancer research has centered on encapsulating genes, RNA therapeutics, or aptamers for targeted delivery. Zhao et al. investigated a pH-responsive peptide (DPRP) anchored in the liposome’s surface for anticancer therapy. Using the thin film hydration method, liposomes were synthesized from DOPE, CHEMS, and CHOL (56:22:22). This formulation displayed an increased particle size, reaching 211 nm in diameter, a narrow PDI, and a negative surface charge. Designed for the co-delivery of polo-like kinase-1-specific siRNA and docetaxel, the system achieved remarkably high encapsulation rates. The key factor enhancing its therapeutic efficacy is its pH-responsive drug release mechanism. The imine bond of DPRP exhibited sensitivity to the tumor cell acidic environment while maintaining high stability under physiological conditions. With its pH-sensitive release and high encapsulation efficiency, this formulation stands out as a potential breakthrough in cancer nanomedicine [40]. Likewise, Du et al. developed pH-sensitive liposomes with a dual role, enhancing tumor immunogenicity and downregulating PD-L1 expression. The researchers used a combination of DSPE-PEG2000, DOPE, and CHEMS (0.5:6:4) to form PEG-modified liposomes via a thin film hydration method, alongside PEI-elastase and PD-L1 siRNA, to prepare PEI-elastase through a Michael addition reaction and an amide coupling reaction using an MHSu linker. The resulting formulation exhibited a large diameter of approximately 175 nm and a negative ζ-potential. In vivo studies on mice confirmed that PEI-elastase exhibited pH-triggered release, with the release being notably higher at pH values of 6.5–7.0. These findings highlight its potential as an effective gene-delivery material addressed for synergistic anti-tumor activities [41]. Similarly, Abuhelal et al. designed a novel nanocarrier composed of a pH-responsive peptide (LAH4-L1) combined with PEGylated cationic liposomes. This formulation encapsulated siRNA, achieving an encapsulation efficacy, which exceeded 90%, demonstrated enhanced gene silencing efficiency in cells, and improved tumor accumulation of labeled siRNA after the application of focused ultrasound in vivo. The liposomes were synthesized using Dioleoyl-dimethylammonium-glutaryl (DODAG), DOPC, CHOL, and MeO-PEG2000-DSPE (2:5.5:2:0.1 or 0.5) along with 4-(2-hydroxyethyl)-1-piperazineethanesulfonic acid (HEPES) and chloroform, utilizing lipid film hydration, five freeze–thaw cycles, and extrusion methods, resulting in a size of approximately 115 nm and a surface charge of around +30 mV. Even though significant siRNA delivery to the tumor was achieved, the effect of siRNA-increased dose is not yet studied [42].

In line with this, Ding et al. developed pH-sensitive liposomal nanocarriers for anticancer therapy, evaluating them in vivo and in vitro. Liposomes, composed of egg PC, CHEMS, and CHOL (33:13:5), were prepared via the film hydration technique and then modified with single-stranded DNA aptamer AS1411 and gold nanoparticles. They were loaded with morin hydrate, achieving high encapsulation efficiency, a large hydrodynamic diameter, and a positive ζ-potential. This novel formulation exhibited pH-responsive drug release, enhancing targeted delivery and therapeutic efficacy. Notably, morin hydrate release was fivefold higher in the tumor’s acidic microenvironment compared to alkaline conditions. Despite its promising results, limitations include difficulty in large-scale production and high associated costs [43]. Expanding the research of pH/redox dual-responsive nanoparticles, Yang et al. utilized them as an siRNA delivery tool, targeting breast cancer cells. To protect siRNA from enzymatic degradation within the body and enable efficient delivery to the target, they used PE, CHEMS, and CHOL (60:40:26, *w*/*w*) to form liposomes via ultrasonic hydration and coated them with a lipid bilayer followed by modification with HA. These nanocarriers, loaded with P-gp siRNA and doxorubicin, achieved very high encapsulation efficacy and loading capacity rates and exhibited a diameter of 210 nm with a negative surface charge. Furthermore, it was observed that as GSH concentration increased from 0 to 10 mM and pH conditions became more acidic, the drug release rate was higher, confirming their redox- and pH-sensitive drug release behavior. Further investigation is required to understand the mechanism of action at a genetic level, particularly how it penetrates multiple barriers in the body to reach the tumor site and exert its effects [44]. Building on multiple research findings, Lafi et al. designed and developed PEGylated pH-sensitive liposomes with an anti-nucleolin aptamer encapsulating γ-cyclodextrin with echinomycin for targeting breast and lung cancer cells. For this purpose, DPPC, DOPE, cholesterol, DSPE-PEG2000 (40:30:30:3), oleic acid, and Lissamine were purchased. Using the thin film hydration technique followed by lyophilization, liposomes were formed with a diameter of approximately 152 nm, a PDI of 0.15, and a negative ζ-potential. The loaded echinomycin (quinomycin A) achieved an encapsulation efficacy of around 4.6% and a loading capacity of 0.025%, showing a slow and stable release at different pH levels, with a higher release at acidic pH (47% at pH = 5.4). These promising findings lack information on the mechanism of action, emphasizing the need for future in vivo and mechanistic studies [45].

**Figure 4:**
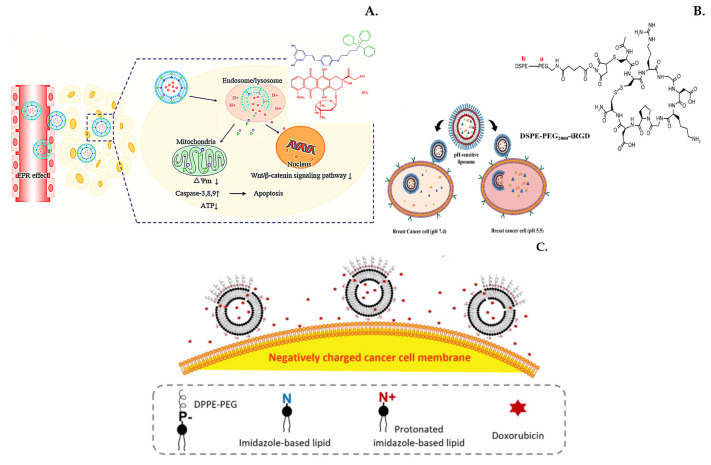
Targeting strategies of pH-responsive liposomes in anticancer therapy: (**A**) Schematic of pH-sensitive liposome encapsulated with Dox and TPP-Res (Dox/TPP-Res), with passive targeting ability via enhanced permeability and retention (EPR) effect, followed by endosomal escape towards mitochondrial targeting, in order to reverse chemotherapeutic resistance in cancer stem-like cells. Adapted from [46]. (**B**) Schematic of αvβ3 integrin and folate-targeted pH-sensitive liposomes with dual ligand modification for metastatic breast cancer treatment via active targeting with targeting ligands on their surface, adapted from [20]. (**C**) Schematic of imidazole-based convertible pH-sensitive liposomes turning from stealth liposomes into cationic liposomes in acidic tumor interstitium. P-, negatively charged phosphate group in DPPE-PEG; N/N+, basic amine in imidazole-based lipids, adapted from [34].

Cancer stem-like cells (CSLCs) are one of the main problems in tumor treatment owing to high tumorigenicity and chemotherapy resistance. Ba et al. synthesized a novel mitochondria-targeted derivative, triphentlphosphonium-resveratrol (TPP-Res), and simultaneously encapsulated it with doxorubicin (Dox) in pH-sensitive liposomes to reverse chemotherapeutic resistance of CSLCs (Figure 4). The cytotoxicity assay showed that the optimal synergistic effect was the drug ratio of 1:1 for TPP-Res and Dox. Cellular uptake and intracellular trafficking assays indicated that the prepared liposomes could release drugs in acidic endosomes, followed by mitochondrial targeting of TPP-Res and nucleus transport for Dox, while the in vivo assay results demonstrated that the constructed liposomes could efficiently accumulate in the tumor region and possess excellent antineoplastic activity in an orthotopic xenograft tumor model with no evident systemic toxicity [46].

Some representative examples of the above-described pH-sensitive targeting strategies of liposomes used in anticancer therapy are illustrated in Figure 4.

A summary of the aforementioned literature case studies regarding the treatment of cancer is also presented in Table 1. In the majority of cases, potent anticancer APIs are incorporated into pH-responsive liposomes. The thin-film hydration method is used as the formulation preparation protocol. The prepared liposomes are evaluated in preclinical studies using various types of cancer cell lines. In all cases, the incorporation of anticancer drugs into pH-responsive liposomes results in an increased therapeutic index, as the same pharmacological effect is achieved at lower concentrations of the anticancer API. Additionally, the incorporation index is quite high, in several cases approaching 100%. Finally, release only at the cancerous tumor is achieved with the use of this highly promising technology.

#### 3.2.2. Gene Delivery and Targeting

pH-responsive liposomes have emerged as a promising tool in gene therapy due to their ability to deliver specific therapeutic agents. Ramírez-Acosta et al. developed nanobioconjugates/magneto-liposomes, composed of soy lecithin, chloroform, and magnetite/silver-pDMAEMA-PEA-BUFII, that could function as pH-responsive polymeric vehicles for the transport and release of circular DNA. The formulation process involved lipid bilayer hydration and sonication, yielding large, uniform liposomes with high stability, suitable for drug delivery. Encapsulation of 3 μg of DNA demonstrated a low but effective loading capacity, with release occurring in a pH- triggered manner. However, delivery efficiency remained limited, at 8.1%, underscoring the need for further optimization at both pre-clinical and clinical scales [47]. On the other hand, Xi et al. designed pH-responsive liposomes for treating acute spinal cord injury. Their multifaceted mechanism of action involved modulating immune cell subtypes to down-regulate acute inflammation, reducing scar tissue formation, promoting angiogenesis and neural differentiation at the injury site, and enhancing functional recovery in vivo. Researchers synthesized liposomes using lecithin, CHOL, and DSPE-PEG-CHO (160:40:4, *w*/*w*%) via the reverse evaporation technique. Trichloromethane and octadecylamine were added to confer a positive charge surface. The formulation achieved high encapsulation efficacy (75%) of IL-4 plasmid (pDNA), forming large high homogeneous particles. It exhibited sustained pH-triggered drug release, increasing as pH became more acidic. Additional in vivo studies are required to verify the mechanism and confirm its therapeutic efficacy [48].

**Table 1:** pH-responsive liposomes intended for anticancer treatment.

Formulation	API	Therapeutic Indication	Preparation Protocol	EE	Characteristics	Release Profile	Added Value	Reference
DOPE, CHEMS, DSPE-PEG2000	cisplatin	cancer	thin-film hydration	EE = 61.23 ± 1.98%	Dh = 191.2 ± 1.67 nm PDI = 0.386 ± 0.009 ζ-potential = −22.5 ± 0.38 mV	Korsmeyer- Peppas model, pH triggered release: <40% at pH = 7.4, 65% at pH = 6.5, >80% at pH = 5.5	anticancer therapy	[8]
DOPE, CHEMS, DSPE-PEG2000/DSPE-PEG2000-Fol	irrinotecan	colorectal cancer	lipid film hydration, size calibration (extrusion)	EE = 62.3 ± 0.9 %	Dh = 165.2 ± 3.1 nm ζ-potential = −9.1 ± 1.2 mV PDI = 0.12 ± 0.08	Controlled release in tumor’s acidic environment	folate-coated pH-sensitive liposomes for anticancer therapy (colorectal cancer)	[18]
DPPC, DSPE-PEG-NH2, /CHEMS, PEG bis(amine), Folate	doxycycline and docetaxel	non-small cell lung cancer	thin-film hydration	EE = 60.1 ± 1.1%	Dh = 82.2 ± 2.4 nm ζ-potential = −0.27 ± 0.031 mV at pH = 7.4, −0.17 ± 0.081 mV at pH = 6.5, 2.94 ± 0.075 mV at pH = 4.0 spherical structure	Targeting FRβ and controlled pH dependent drug release: 42% for docetaxel and 25% for doxycycline at pH = 7.4, 61% for docetaxel and 39% for doxycycline at pH = 6.5, 99% for docetaxel and 78% for doxycycline at pH = 4.0	FRβ-targeted pH-sensitive liposomes for early diagnosis and treatment of non-small cell lung cancer (NSCLC)	[19]
phosphatidylocholine (E80), DOPE, CHEMS, DSPE-PEG2000-COOH, folic acid and iRGD surface modifications	fluorouracil	breast cancer	thin-film hydration	EE = 93.1 ± 2.58%	Dh = 152 ± 3.27 nm ζ-potential = −14.8 ± 2.8 mV PDI = 0.22 ± 0.2	pH-triggered release at 24, 48 and 72 h: 30.0 ± 3.7%, 38.7 ± 3.9%, 46.4 ± 5.7%, at pH = 7.4, 68.3 ± 3.7%, 83.3 ± 4.3%, 84.5 ± 6.2% at pH = 5.5	5-Fluorouracil (5-FU)-loaded folic acid (FA) and iRGD (peptide) surface-modified pHLips (FA-iRGD-5-FU-pHLips) for breast cancer treatment	[20]
DPPC, cholesterol, DDAB, DG-CDP	NH2-PEGylated gold nanoparticles	ovarian cancer cells	thin-film hydration, extrusion	EE = 78 ± 4 %	Dh = 651 ± 9 nm ζ-potential = −6.6 ± 0.3 mV spherical shapes, PDI = 0.33 ± 0.01	zero order model, Peppas model or the Higuchi model, controlled release at acidic pH and hyperthermia conditions	nanocarrier with a self-targeted component, a dual pH/thermo- responsive behavior and functionalized surface which allows preventing the immune system, recommended for photothermal therapy	[22]
DOPE, CHEMS, DSPE-PEG2000 and DSPE-PEG2000-DVar7	doxorubicin	highly malignant tumors	thin-film hydration and filtration	EE = 93%	Dh = 130 nm PDI < 0.2	pH triggered release in acidic environment, at pH = 5.3 the drug’s release was five times higher than at pH = 7.4, prolonged drug release	DVar7 peptide-based dual pH-responsive liposome with acidity regulation (injection of glycose in tumor environment) for anticancer therapy—in vivo experiments/potential usage in more malignant tumor models	[23]
DOPE, CHEMS, DSPE-PEG2000/HSPC	Doxorubicin, Chloroquine, E64d	cervical cancer	lipid film hydration, extrusion, ultracentrifugation			SpHL-DOX has a faster intracellular release when compared to nSpHL-DOX but promoted sustained release if compared to free-DOX	mechanism of intracellular release from pH-sensitive liposomes using cervical cancer HeLa cells (viability, internalization, intracellular trafficking and delivery) +cell death mechanism/ lysosome acidification and protease activity (using chloroquine and E64d) enhance drug release	[24]
DOTAP and cholesterol	all-trans retinoic acid (ATRA)	lung cancer	thin-film hydration method, ultracentrifugation, extrusion	EE = 93.7 ± 3.6%	Dh = 231 ± 2.35 nm ζ-potential = 6.4 ± 1.19 mV	gradual/steady release kinetics	cationic pH-responsive liposome for anticancer therapy	[25]
EPC, PDMAEMA-b-PLMA	TRAM-34	glioblastoma	thin-film hydration method	EE1 = 73% EE2 = 62%	1 (9:0.5:1.75): Dh = 164.4 ± 8.0 nm PDI = 0.236 ± 0.052 ζ-potential = 14.0 ± 1.2 mV 2 (9:0.5:2.45): Dh = 139.2 ± 0.8 nm PDI = 0.193 ± 0.006 ζ-potential = 12.4 ± 0.8 mV	pH-responsive drug release, 1: 40% after 1 h, 55% after 6 h, 2: 30% after 1 h, 37% after 5 h/ in acidic conditions: 1: 70% after 15 min followed by 80%, 2: 85% after 30 min, ending up at almost 100% after 1 h for 1, 2	pH-responsive liposomes for the treatment of glioblastoma	[26]
DOPE, DSPE-PEG2000, CHEMS	pirfenidone	idiopathic pulmonary fibrosis	ammonium sulfate gradient method, calcium acetate gradient method	EE = 86.65 ± 1.36%	Dh = 106.6 ± 1.36% PDI = 0.061 ± 0.001 ζ-potential = −74 mV morphology: quasi-spherical	rapid release at acidic pH, 80% of the drug was released during 24 h, long-lasting release, the action time of drug could be prolonged by PSLs	pH-sensitive PEGylated liposome	[27]
PC and 3-methylglutarylated-dextran residues (Mglu-Dex)	sorafenib and hemin	antigen delivery and immune modulation					pH-sensitive hybrid liposomal vesicle (liposomes +amphiphilic dendrimers for TME, for antigen delivery and immunity	[28]
DOPA, DSPE-PEG5000 and carbonic anydrase inhibitor coating	carbonic anhydrase inhibitor (CAI)	anticancer immunotherapy	one pot method, thin film dispersion–membrane extrusion technique	EE = 61.7 ± 1.1%	Dh = 115.5 ± 14.0 nm	at pH = 5.8 90.8 ± 3.8% Ca^2+^ 87.6 ± 3.3% CAI at pH = 7.4 32.0 ± 3.9% Ca^2+^ 51.2 ± 9.5% CAI	pH-responsive liposome-coated carbonic anhydrase inhibitor (CAI)-loaded calcium carbonate nanoparticle in anticancer radio-immunotherapy as it causes calcium death and strengthen the immune responses	[29]
CHEMS, DSPE-mPEG2000-COOH, DOPE	RA-V, PD-A/PD-L1 inhibitor	colorectal cancer therapy	thin film hydration method, ultrasonic treatment, extrusion	EE = 80.1% (for RA-V) EE = 79.6% (for BMS)	Dh = 192.46 ± 7.91 nm ζ-potential = −8.71 ± 0.60 mV spherical morphology	controlled drug release in the intracellular acidic environments	Liposome in anti-tumor immunity as cancer cell camouflage	[30]
lecithin and PEG-OE-L	Chlorine e6 (Ce6), paclitaxel and doxorubicin	many cancer types	thin film ultrasonic dispersion method	EE = 92.27% (for paclitaxel) and EE = 90.34% (for Ce6)	Dh = 58.23 nm ζ-potential = −45.45 to −28.25 mV morphology: spherical	rapid drug release and enhanced endocytosis in a slightly acidic environment in light irradiation at 660 nm/ without light exposure/with light protection: 30.84% (for Ce6) and 28.64% for PTX at pH = 7.4, 56.44% (for Ce6) and 52.27% (for PTX) at pH = 6.5, under laser irradiation: 62.48% (for Ce6) and 56.33% (for PTX) at pH = 7.4, 78.12% ( for Ce6) and 75.17% (for PTX) at pH = 6.5	pH and photo dual-responsive liposomes for targeted anti-tumor activity	[31]
SN38-PA, LA-SN38 and Di-SN38-PC	Moeixitecan, transferrin, TAT-PEG-SN38, survivin siRNA	colon cancer	film dispersion technique			prolonged release, reversible pH-dependent hydrolysis of lactone ring (at pH = 7.4 the lactone ring partially hydrolyzes to a carboxylate form without having any therapeutic effect, at pH = 9 the lactone ring is hydrolyzed and SN38 only exists in the carboxylate form)	SN38-based drug delivery systems for anticancer therapy with stronger cytotoxicity	[32]
cholesterol, soy lecithin, DSPE-PEG2000, C18-TAT peptide and cinnamaldehyde	Pt (IV) prodrug disuccinatocisplatin (DSCP) and cinnamaldehyde (CA)	many cancer types	lyophilization (DSCP), self-emulsifying solvent evaporation + thin film hydration method (DLCP liposomes)		Dh = 155 ± 3.6 nm ζ-potential = −21.5 ± 0.5 mV PDI < 0.4	CA release: 38.96% at pH = 7.4, 63.02% at pH = 5.5 DSCP release: 36.26% at pH = 7.4, 59.26% at pH = 5.5	TME-/+ pH-responsive liposome enhancing intracellular ROS activates the apoptotic pathway of tumor cells (synergistic strategy in anticancer therapy)	[33]
lipids that carry imidazole-based headgroups, phosphatidylethanolamine-polyethylene glycol	doxorubicin	solid tumors	thin-film hydration, freeze–thaw cycles, extrusion	EE = 89.86 ± 1.27	Dh = 128.1 ± 8.3 nm PDI = 0.078 ± 0.021	pH-triggered release, >50% at pH = 6.0 in 6 h for DHMI lipid	pH-sensitive PEGylated convertible liposomes with imidazole-based lipids (DHMI)	[34]
DPPC, cholesterol and DSPE-PEOz2000	IR780 as a sonosensitizer and metformin	sonodynamic therapy (SDT)	thin-film hydration	LE = 18% of metformin, 84% of IR780	Dh = 220.193 nm (at pH = 5 the size became much smaller) ζ-potential = −33.69 mV PDI = 0.165	metformin had a pH- triggered release: 49.7% at pH = 7.4, 64.13% at pH = 6.5, 95% at pH = 5.5 IR780 did not, 18.39% at pH = 7.4 and at more acidic pH the release was slightly accelerated	pH-responsive drug-loaded liposomes to reduce oxygen consumption, attenuate hypoxia-induced resistance to SDT, and improve the SDT	[35]
cholesterol, DSPE-Hyd-PEG2k, lecithin and DSPE	dermaseptin-PP (65)	lung cancer	thin-film hydration	EE = 86.26 ± 0.19%	Dh = 96.78 ± 1.50 nm ζ-potential = 6.94 ± 0.58 mV PDI = 0.15 ± 0.02	the cellular uptake increased with the decrease in pH (at pH = 5 was x2 higher than that at pH = 7.4	pH-responsive liposome encapsulates an a-helix anticancer peptide to enhance efficacy and reduce risk hemolysis in lung cancer	[36]
lecithin (egg yolk) and cholesterol, modified with ginsenoside and hyaluronic acid	paclitaxel	HCT-116 cells	thin-film dispersion technique	EE = 95.31 ± 1.41%	Dh = 188.50 ± 0.40 nm PDI = 0.28 ± 0.01 ζ-potential = −9.00 ± 0.48 mV	rapid release within the first 5 h, followed by a slower release from the 5th to the 10th hour, ultimately stabilizing at the 12th hour/ pH triggered drug release: 35% at pH = 7.4, 95% at pH = 5.0	ginsenoside compound K(CK)/HA—modified liposomes for anticancer therapy	[37]
DSPE-PEG-NH2, DOPE and N-azidoacetylgalactosamine tetraacylated (Ac4GalNA),	Rhodamin as marker	many cancer types	thin-film hydration	EE = 36.4%	Dh = 164 nm ζ-potential = –12.4 mV	pH triggered release: 72% at pH = 5, 29% at pH = 7.4 after 12 h	pH-responsive azidosugar liposomes camouflaged with natural cancer-cell membrane for tumor cell-selective glycan engineering (reduced protein corona formulation and admirable phagocytosis by macrophages)	[38]
DOPE, CHEMS, DSPC and mPEG2000-DSPE	cytotoxin (CTX)	many cancer types	thin-film hydration	EE = 51.78%	Dh = 127.92 ± 3.75 nm ζ-potential = −23.33 ± 0.67 mV PDI = 15.00 ± 1.91 morphology: spherical	sustained and controlled release in tumor acidic microenvironment, in the first 12 h: no significant difference in release profile at different pH values after 72 h: at pH = 5.5 95.69% was released, at pH 7.4 27.17% was released	pH-responsive liposomes with high CTX load for targeted acidic stimuli release in tumor microenvironment	[39]
DOPE, CHEMS, cholesterol and pH-responsive peptide (DPRP)	gene, siRNA, docetaxel, aptamers	many cancer types	thin-film hydration	EE = 95.9 ± 0.52%	Dh = 211.3 ± 1.6 nm PDI = 0.219 ± 0.012 ζ-potential = −36.1 ± 1.46 mV	the imine bond of DPRP exhibited a pH response in the tumor cell environment and maintained high stability under physiological conditions	pH-responsive peptide (DPRP) anchored in liposome’s surface for anticancer therapy	[40]
DSPE-PEG2000, DOPE, CHEMS and PEI-elastase	PD-L1 siRNA	many cancer types	thin-film hydration		Dh = 175.7 ± 10.2 nm ζ-potential = −9.15 ± 1.6 mV	higher release at pH = 6.5–7.0	PEI-elastase/PD-L1siRNA encapsulated in (PEG-modified) pH-sensitive liposomes for enhancing tumor immunogenicity and downregulating PD-L1 expression	[41]
DODAG, DOPC, cholesterol, MeO-PEG2000-DSPE and pH-responsive peptide (LAH4-L1)	siRNA	many cancer types	lipid film hydration and five freeze–thaw cycles, extrusion	EE > 90%	Dh = 115 ± 10 nm ζ-potential = +29.0 ± 2.3 mV		pH-responsive peptide (LAH4-L1) combined with PEGylated cationic liposome showed improved gene silencing efficiency in cells and general labeled siRNA tumor accumulation (after using focused ultrasound FUS)	[42]
egg PC, CHEMS and cholesterol, modified with single-stranded DNA aptamer AS1411 and gold nanoparticles	Morin hydrate	many cancer types	film hydration	EE = 89.6%	Dh = 150 nm ζ-potential = 36.4 ± 0.3 mV	pH responsive drug release: 54% at pH = 5.0, 10% at pH = 7.4	pH-sensitive liposomes modified with single-stranded DNA aptamer AS1411 and gold nanoparticles for anticancer therapy (in vitro + in vivo)	[43]
PE, CHEMS and cholesterol, modified with hyaluronic acid	P-gp siRNA and doxorubicin	breast cancer	ultrasonic hydration	EE = 92.98%	Dh = 210 nm ζ-potential = −45 mV	GSH-dependent (6% to 62% as GSH ‘s concentration rose from 0 to 10 mM/redox sensitive behavior) + pH-sensitive release (78% at pH = 7.4, 89% at pH = 5)	pH/redox dual responsive nanocarrier system (coated by lipid bilayer and modified with HA) as a siRNA delivery tool targeting breast cancer cells (to protect siRNA enzymatic degradation within the body and enable efficient delivery to target)	[44]
DPPC, DOPE, cholesterol, DSPE-PEG2000, oleic acid and Lissamine, with an anti-nucleolin aptamer	γ-cyclodextrin with echinomycin	breast and lung cancer	thin-film hydration, lyophilization	EE = 4.6 ± 0.53%	Dh = 152 ± 11 nm PDI = 0.15 ± 0.01 ζ-potential = −28 ± 3.4 mV	pH triggered release: 8.82 ± 2.5% at pH = 7.4, 46.65 ± 6.3% at pH = 5.4 slow and stable release at different pHs	PEGylated pH-sensitive liposome with anti-nucleolin aptamer encapsulates γ-cyclodextrin with echinomycin (AptNCL-PEGLippH-ECgCD) in anticancer therapy	[45]
DOPE, HSPC, CHEMS and cholesterol	triphentlphosphonium-resveratrol (TPP-Res), doxorubicin	cancer stem-like cells	thin-film hydration, ammonium sulfate gradient method, extrusion	EE = 94.5 ± 0.32% for doxorubicin EE = 68.5 ± 1.74% for TPP-Res	Dh = 165.2 ± 5.7 nm ζ-potential = −19.2 mV	similar steady, sustained pH-responsive release: 35% at pH = 7.4, 65% at pH = 5.0	pH-responsive liposome combined with mitochondria-targeted derivate TPP-Res show effective endo-lysosomal escape, mitochondrial targeting by decreasing mitochondrial membrane potential, activating caspase, reducing ATP level and suppressing the Wnt/β-catenin pathway	[46]

#### 3.2.3. Bacterial Infections

Research by Deiss-Yehiely et al. and others has expanded the use of pH-responsive liposomes into bacterial infections, offering a breakthrough in biofilm-targeted drug delivery. On the one hand, using the thin film hydration method, liposomes composed of CHOL, DSPC, and DSPG (34:33:33) were formed and coated via the layer-by-layer technique with polycationic poly-L-lysine and polyanion 1–5. These polymers undergo rapid hydrolysis in acidic biofilm environments, converting the surface charge from negative to positive, enabling deeper biofilm penetration. The resulting pH-responsive nanocarriers loaded with tobramycin achieved great loading capacities targeted Pseudomonas aeruginosa infections. They exhibited small sizes (Dh = 117 nm), narrow polydispersity, and a large positive ζ-potential, enhancing drug delivery and biofilm penetration. These promising findings have yet to be explored for treating infections caused by other microbial species [49]. On the other hand, Wang et al. investigated the differences between alternative formulations of liposomes capable of self-targeting tumor sites and infectious biofilms. Liposome one was composed of DCPA and H_2_O, liposome two of DPPC, and liposome three of DCPM, and they were loaded with ciprofloxacin and rhodamine. All liposomes exhibited a spherical morphology with a diameter of 100 nm and a ζ-potential ranging from neutral to slightly positive, depending on pH variations. Liposome one was distinguished, highlighting the superiority of water as a pH-responsive functional component. Furthermore, while all demonstrated long blood circulation times and rapid self-targeting to the acidic environment, liposome one surpassed them, exhibiting full blood compatibility with better blood circulation times, resulting in better therapeutic efficacy while targeting an infectious biofilm of *St. aureus* and *Myc. Tuberculosis*. Given its significance, this formulation necessitates further research in the context of metastasis, other infection sites, or solid tumors [50]. A separate research team, Luo et al., designed hybrid nanoparticles for anti-infection of bacteria to treat acute lung infection (ALI). They used DSPG and CHOL (3:1) in a chloroform, methanol, and water mixture (60:32:8, *v*/*v*), through lipid film hydration, extrusion, and layer-by-layer processes, to form liposomes, followed by coating them with PBAE/NaAlg. They exhibited homogeneous spherical shapes with a diameter of around 200 nm, a narrow PDI, and a negative surface charge. The resulting liposome–polymer hybrid NPs were loaded with streptomycin via the pH-gradient method, achieving an encapsulation efficacy of approximately 25% and a remarkably high loading capacity of 490%. Streptomycin release exhibited a prolonged pH-triggered profile, with 90% release after 10 h and nearly 100% after 24 h at pH = 6.0. These promising formulations demonstrate effective drug delivery; however, further investigation and scalability studies are needed [51].

#### 3.2.4. Nanovaccines

The applications of pH-responsive liposomes extend into vaccination strategies, as demonstrated by Szachniewicz et al., who explored their potential and developed pH-sensitive liposomes loaded with the triple fusion antigen protein Ag85B-ESAT6-Rv2034 (AER) for use in tuberculosis vaccination. The liposomes, created using the thin film hydration method, were composed of DOPC, EPC, DOPE, and DOBAQ (3:4:5:2). They demonstrated small particle sizes with narrow size distribution and a positive ζ-potential, which indicates a stable surface charge. This formulation showed the best stability and immune responsiveness as a promising vaccine. However, further research is required as regards CD4+ and CD8+ T-cell activation, B-cell responses, and the long-term effects of antigen dose and excipients on vaccine efficacy. Discrepancies with genetic knockout results and the single time point assessment also warrant further investigation into immune response dynamics and long-term protection [52].

A summary of the aforementioned literature case studies regarding the treatment of infections and vaccination is also presented in Table 2.

#### 3.2.5. Diagnosis

It is a well-accepted principle that faster diagnosis contributes to more favorable outcomes. In alignment with this, pH-responsive liposomes have been effectively utilized as advanced diagnostic tools. Kang and Park introduced an innovative portable, rapid, and sensitive method for detecting acetylcholine’s concentration with pH-sensitive liposomes. Using DPPC and DPGS (60:40) or pure DPPC, they synthesized liposomes through the thin film hydration method, achieving a uniform average size of 150 nm, with a distribution range of 130–170 nm and highly positive or negative ζ-potential. The resulting formulation encapsulated potassium ferricyanide, K3Fe[CN]6, which was released in a pH-triggered manner. Upon further analysis, the hydrolysis of Ach produced acetic acid and choline, reducing the pH value. This pH reduction destabilized the liposomes, triggering the release of potassium ferricyanide. However, concerns remain regarding the formulation’s resistance and proper storage conditions to maintain efficacy and prevent degradation over time. Last but not least, a compatible, user-friendly device should be designed for daily use to enable faster diagnosis and improve prognosis [53]. Other researchers have successfully integrated early diagnosis and therapeutic functions through pH-sensitive liposomal formulations. Sia et al. designed a dual pH- and magnetic–responsive Pickering emulsion as a novel formulation of nanoparticles (SPION) encapsulated in pH- and magnetic–responsive liposomes. Magneto-liposomes (MLP) in a Pickering emulsifier are biocompatible, nontoxic, and capable of targeting several sites via remote control. Super paramagnetic iron oxide nanoparticles were prepared and loaded in liposomes composed of L-a-phosphatidylcholine, CHOL (6:1), chloroform, and PEG3000 and assembled through thin film hydration, extrusion, and low-energy mixing (liposome-stabilized Pickering emulsion) methods. SPIONs demonstrated small size (Dh = 8.8 ± 1.2 nm), negative surface charge, and significant magnetic properties. MLPs showed large size (Dh = 176.8 ± 6.8 nm) and strong negative surface charge at neutral and mildly acidic pH. Their stability was studied, indicating high rates at pH > 7.4 (30% MLP concentration, 10% *v*/*v* oil loading) and for 21 days at a temperature < Tm of MLP. However, scaling up remains a challenge [54]. Furthermore, Taiying Chen et al. presented a nanoplatform with pH-responsive charge–reversal properties, an ability to alleviate tumor microenvironment hypoxia, and an ability to contribute to photodynamic therapy for diagnosis and treatment of hepatocellular carcinoma (HCC). For this purpose, Fingolimod (FTY720) was encapsulated in liposomes composed of DSPE-PEG-PEI-DMA, DPPC, CHEMS, and DSPE-PEG Mal (1:1:1:1), synthesized by the hydration film method, coupling reaction, sonication, and extrusion methods. The resulting nanoplatform exhibited a spherical, yolk shell-like structure around 50–100 nm and a ζ-potential that could change from slightly negative to slightly positive depending on pH variations. This formulation was tested under different conditions, indicating sustained and controllable drug release in acidic, H_2_O_2_, and near-infrared environments, which confirmed its promising potential. However, the process of scaling up remains unexplored [55].

#### 3.2.6. Other Applications

Expanding the application of pH-responsive liposomes, Feng et al. investigated an innovative method to prevent the deterioration of blueberries during storage and transportation. They developed pH-sensitive liposomes covered in a film with controlled release properties and oxidation resistance. For this purpose, they used Tween-80, high-potency lecithin (PC), CHOL, and DPPH (CHOL/PC with 30 wt% and TP/CHOL-PC with 5 wt%), and through thin-film dispersion and drop-casting methods, liposomes with a diameter of approximately 810 nm and negative ζ-potential were constructed. Tea polyphenols (TP), sodium alginate (SA), and carboxymethylcellulose (CMC) were encapsulated (EE = 61%) into the resulting formulations and were released in a pH-triggered way (23.07% at pH = 6, 41.08% at pH = 3). This form of packaging shows great potential for applications in other fruits or vegetables and could be adapted for various produce types by integrating different polysaccharides and antioxidants, thyme, or oregano [56].

## 4. Trends and Challenges for the Design and Development of pH-Responsive Liposomes

### 4.1. Formulation Trends

As mentioned above, several techniques are used to design and develop pH-responsive liposomes. In the majority of cases, the thin-film hydration method is chosen for the laboratory preparation of pH-responsive liposomes since it is very easy. Sonication, extrusion through filters, and freeze–thaw are used as size reduction methods. Recently, hybrid polymer-grafted liposomes with pH-responsive drug release properties were formulated using controlled microfluidics. This technique gave the opportunity to control the crucial formulation parameters, including the flow rate ratio (FRR) and the total flow rate (TFR). FRR is of paramount importance for the collective injection speed of both phases through the microfluidic chip, while FTR is for the proportion between the aqueous and lipid phases. The microfluidic techniques increased the storage stability of the prepared systems for three months, which is very promising for the scale-up of the pharmaceutical formulation [57]. Additionally, some authors underlined that for the pH-responsive liposomes, some main ingredients are the building blocks for their applications in drug and gene delivery and targeting [58,59]. Namely, one ionizable lipid, one or more helper phospholipids, cholesterol, and a pegylated lipid are required to achieve endosomal escape and/or pH-responsive release of the encapsulated cargo. Last but not least, dual responsive liposomes are a promising formulation strategy against cancer. Wang et al. [60] developed a liposome-functionalized photothermal agent with pH responsiveness. In this case, DOPE changed its molecular crystalline form under low pH values and led to pH-triggered release, while Efimova et al. [61] developed a pH-sensitive multiliposomal container for encapsulation and controlled drug release, combining PEGylated cationic and anionic vesicles to manage quick responses to small pH variations and, thus, controlling the kinetics of cargo release.

In general, the current formulation strategies of the pH-sensitive liposomes are mostly directed to increase the precision of the targeting, with exploitation of many different categories of functional biomaterials, like peptides, antibodies, targeting ligands like folate, customized pH-sensitive polymers, etc., along with increasing their stealth properties and fine-tuning the triggers, for example, with PEGylation protective layers to balance their circulation longevity in acidic environments towards effective intracellular delivery. Furthermore, the combination of smart structural design strategies, like multi-component delivery vehicles and multi-layered or polymer-integrated liposomes that combine robustness with responsive release profiles, along with other stimuli-type modalities, like temperature sensitivity, are currently being investigated [62].

PEGylation is a strategy to increase the circulation time of liposomes and imparts “stealth” properties but also influences the pH responsiveness [63,64,65]. Firstly, it is well-established that the PEG length influences the stability of the phospholipid membrane, the physicochemical stability, and the interactions of the serum proteins of liposomes. The liposomal nanomedicines, i.e., Doxil^®^, generally use PEG with a molecular weight of 2000, while the PEG chains with lengths between 2000 and 5000 are found to exhibit stealth properties, too. The distribution and pharmacological performance of encapsulated API are also influenced by the PEG length. According to Lim et al. [63], the increased length of PEG reduces protein adsorption and thereby maximizes stealthiness. For the pH-responsive liposomes, according to recent published data, it is highlighted that PEG stabilizes DOPE lipidic bilayers into the formation of the lamellar phase by increasing the area per lipid through penetration into the bilayer [64]. Since the optimal PEG length should be evaluated taking into account the encapsulated API, the target, the administration route, and the desired drug release rate, it seems that the PEG with a molecular weight of 2000 at a 2–5% weight ratio can be the starting point for the formulation of PEGylated pH-responsive liposomes [65].

On the other hand, PEGylation is also synonymous in some cases with accelerated blood clearance, formation of anti-PEG antibodies, hypersensitivity, and complement activation-related pseudoallergy [66,67]. The addition of PEGylated lipids may cause the aforementioned adverse effects in the cases of administration of pH-responsive liposomes, especially after repeated doses in chronic diseases. Generally, as it is discussed in the literature (Table 1), the pH-responsive liposomes do not exhibit cytotoxicity, and reduce the toxicity of anticancer APIs [68].

### 4.2. Formulation Challenges

The central formulation goal is to achieve pH responsiveness and the endosomal escape of the prepared liposomes. The conditions of the microenvironment of the cancer, which is synonymous with human cancers’ heterogeneous extracellular matrix, especially the pH, vary in the different types of tumors, making the studies of the formulation scientists quite challenging [69]. Furthermore, several investigations have appeared in the literature with many formulations of pH-responsive liposomes and other nanocarriers, but only a few preclinical and clinical data showed real endosomal escape. For this reason, early research is focused on branched endosomal disruptor (BEND) lipids mediating delivery of mRNA [70]. Other challenges of the pH-responsive liposomes are the physicochemical stability and scalability, which are the main gaps for their fast clinical translation. GMP-compliant, reproducible, and scalable manufacturing processes are crucial to be established and validated. For example, any process modification following liposomal product marketing, as in the products need to be diluted or reconstituted, requires comparative investigations. Therefore, it is advisable to avoid changes in liposomal manufacturing after final approval. These challenges appear in all liposomal formulations, i.e., conventional, pegylated, and others. However, there is a growing interest from regulatory agencies in streamlining industrial translation of these products. The cost of the liposomes, especially for those with ionizable and pegylated phospholipids and the advanced techniques for their characterization (i.e., cryogenic TEM, AFM, and light scattering techniques, etc.), leads the pharmaceutical industries to adapt other technologies for the development of the candidate pharmaceuticals. The complexity of liposomal medicines coming from the preformulation studies until their production, including the quality control during all the stages of development, is one of the most important barriers in the current pharmaceutical technology [71].

## 5. Conclusions

Stimuli-responsive drug delivery systems, like pH-responsive liposomes, have gained significant attention for their ability to enhance therapeutic efficacy while minimizing toxicity. The development of pH-responsive liposomes represents a promising strategy for carrying both drugs and natural compounds, particularly when combined with other nanocarriers such as gold nanocarriers or polymeric micelles, or when triggered by external stimuli like light, redox conditions, heat, ultrasound, or magnetic fields. Such approaches contribute to more targeted therapy, while PEGylation further prolongs circulation time, resulting in improved systemic stability and optimized therapeutic outcomes. These liposomes are formulated using the thin film hydration method, with lipids such as DOPE, CHEMS, CHOL, DPPC, DOBAQ, EPC, and DOPC. Commonly used solutions in this process include chloroform, methanol, and water in various ratios. The hydrodynamic diameter ranges from 100 nm to 200 nm, which is influenced by surface modifications in liposomal formulation such as aptamers or PEGylation. Their ζ-potential varies corresponding to pH alterations, remaining negative at neutral pH conditions and changing to positive in acidic pH. PEGylated liposomes tend to have more neutral ζ-potential (−5 to −28 mV) in contrast to cationic liposomes with positive charge (+20 to +50 mV). The polydispersity index remains close to zero, indicating a homogenous size distribution. Despite their advantages, pH-responsive liposomes face several limitations that hinder their clinical application.

The limited number of in vivo studies complicates the research on their behavior in complex biological environments. Furthermore, the lack of clinical trials restrains their impact on different populations, giving insufficient information for their therapeutic profile and side effects. Moreover, dose alterations are not usually tested, which further complicates their applicability in personalized medicine, as they can significantly affect drug release, efficacy, and biocompatibility. Stability still remains a pressing concern, as the liposomal membrane may destabilize, provoking degradation, aggregation, or release of the therapeutic compound prematurely. Last but not least, scalability continues to be a challenge, as the production of uniform, stable-in-storage-and-distribution, industrial-scale pH-responsive liposomes could jeopardize safety and therapeutic efficacy. To the best of the authors’ knowledge, there are not any pH-responsive liposomes available in the market, except the vaccines for COVID-19, which use ionizable lipids [72]. The majority of the studies with pH-responsive liposomes are evaluated in preclinical studies and in animal models. The complexity of their formulation (more than three ingredients, except the active substance, and the characterization techniques, etc.), the challenges in scalability, and the regulatory gaps are the main current bottlenecks to the fast clinical translation of pH-responsive liposomes. In conclusion, tackling these difficulties requires advancements in large-scale manufacturing processes and extensive clinical assessments to ensure the safety, efficacy, and stability of these innovative drug delivery systems.

## Figures and Tables

**Table 2:** pH-responsive liposomes intended for antimicrobial treatment and vaccination.

Formulation of pH-Responsive Liposomes	Active Agent	Therapeutic Indication	Reference
cholesterol, DSPC and DSPG, coated with polycationic poly-L-Lysine and polyanion	tobramycin	*Pseudomonas aeruginosa* infections	[49]
DCPA or DPPC or DCPM	ciprofloxacin and rhodamine	*St. aureus* and *Myc. Tuberculosis* infections	[50]
DSPC and cholesterol, coated with PBAE/NaAlg	streptomycin	acute lung infection (ALI) bacteria	[51]
DOPC, EPC, DOPE and DOBAQ	triple fusion antigen protein Ag85B-ESAT6-Rv2034	tuberculosis vaccination	[52]

## Data Availability

No new data were created or analyzed in this study. Data sharing is not applicable to this article.

## Abbreviations

The following abbreviations are used in this manuscript:

CHEMS	Cholesteryl hemisuccinate
CHOL	Cholesterol
DCPM	Dicetylphosphatemethylester
DCPA	Dicaproylphosphatidic acid
DODAG	Dioleoyl-dimethylammonium-glutaryl
DOBA	Dioleoylbenzylamidopropyl-quaternary ammonium
DOPC	Dioleoylphosphatidylcholine
DOPE	Dioleoylphosphatidylethanolamine
DOTAP	Dioleoyltrimethylammonium propane
DPGS	Dipalmitoylphosphatidylglycerol sodium salt
DPPC	Dipalmitoylphosphatidylcholine
DPPE	Dipalmitoylphosphatidylethanolamine
DPPG	Dipalmitoylphosphatidylglycerol
DPPH	Dipalmitoylphosphatidylhydrazide
DSPC	Distearoylphosphatidylcholine
DSPE	Distearoylphosphatidylethanolamine
DSPG	Distearoylphosphatidylglycerol
EPC	Egg phosphatidylcholine
EYPC	Egg yolk phosphatidylcholine
HA	Hyaluronic Acid
HEPES	4-(2-hydroxyethyl)-1-piperazineethanesulfonic acid
HSPC	Hydrogenated soy phosphatidylcholine
MPLA	Monophosphoryl lipid A
OA	Oleic acid
PC	Phosphatidylcholine
PDMA-b-PLMA	Poly(2-(dimethylamino)ethyl methacrylate)-block-poly(lauryl methacrylate)
PDMAEMA-b-PLMA	Poly(N,N-dimethylaminoethyl methacrylate)-block-poly(lauryl methacrylate)
PLGA	Poly(lactic-co-glycolic acid)
POPC	Palmitoyl-oleoyl-phosphatidylcholine
PS	Particle Size
PDI	Polydispersity Index
EE	Entrapment/Encapsulation Efficiency
Dh	Hydrodynamic diamete
